# Correlations between Clinical Features and Mortality in Patients with *Vibrio vulnificus* Infection

**DOI:** 10.1371/journal.pone.0136019

**Published:** 2015-08-14

**Authors:** Hong Zhao, Lichen Xu, Huihui Dong, Jianhua Hu, Hainv Gao, Meifang Yang, Xuan Zhang, Xiaoming Chen, Jun Fan, Weihang Ma

**Affiliations:** State Key Laboratory for Diagnosis and Treatment of Infectious Diseases, the First Affiliated Hospital, School of Medicine, Zhejiang University, Hangzhou, Zhejiang, 310003, China; Beijing Institute of Microbiology and Epidemiology, CHINA

## Abstract

*Vibrio vulnificus* is a common gram-negative bacterium, which might cause morbidity and mortality in patients following consumption of seafood or exposure to seawater in Southeast China. We retrospectively analyzed clinical data of patients with laboratory confirmed *V*. *vulnificus* infection. Twenty one patients were divided into a survival group and a non-surviving (or death) group according to their clinical outcome. Clinical data and measurements were statistically analyzed. Four patients (19.05%) died and five patients gave positive cultures from bile fluid, and 16 other patients gave positive culture from blood or blisters. Ten patients (47.62%) had an underlying liver disease and marine-related events were found in sixteen patients (76.2%). Patients with heavy drinking habits might be at increased mortality (p = 0.028). Clinical manifestations of cellulitis (47.6%), septic shock (42.9%) and multiple organ failure (28.6%) were statistically significant when comparing survivors and non-survivors (p = 0.035, p = 0.021 and p = 0.003, respectively). The laboratory results, including hemoglobin < 9.0 g/L (p = 0.012), platelets < 2.0×10^9^ /L, prothrombin time activity (PTA) <20%, decreased serum creatinine and increased urea nitrogen were statistically significant (p = 0.012, p = 0.003, p = 0.028 and p = 0.028, respectively). Patients may be at a higher risk of mortality under situations where they have a history of habitual heavy alcoholic drink consumption (p = 0.028, OR = 22.5, 95%CI 1.5–335.3), accompanied with cellulitis, shock, multiple organ failure, and laboratory examinations that are complicated by decreased platelets, hemoglobin and significantly prolonged prothrombin time (PT).

## Introduction

*Vibrio vulnificus* is a gram-negative, curved, rod-shaped bacterium that is present in warm seawater and is a member of a group of vibrios that are called halophilic bacteria. Roland *et al*. reported the first clinical case of *V*. *vulnificus* infection (VVI) that presented with leg gangrene after exposure to sea water in 1970 [1]. Additionally, Hollis *et al*. first isolated this bacteria from blood and defined the laboratory characteristics of this pathogen in 1976 [2]. Moreover, the first clinical characteristics and epidemiological description of 39 patients with VVI were reported in 1979 by Blake *et al* [3]. Not until 1979, Farmer and his group named this pathogen as *V*. *vulnificus* [4].

Some individuals might be more easily infected with *V*. *vulnificus* following consumption of contaminated seafood or following exposure of an open wound to seawater, especially in situations where there is underlying chronic liver disease [5]. Healthy individuals infected with *V*. *vulnificus* though ingestion present with fever, chills, vomiting, diarrhea, and abdominal pain. Some immuno-compromised subjects that are infected with this bacterium might present with a severe and life-threatening illness that is characterized by septic shock and blistering skin lesions [5,6].

The hospitalization rate for VVI was more than 80% and the mortality rate was more than 30% in the USA [7]. In China, the mortality rate ranged from approximately 18% to 56% [8–13]. Patients with some additional susceptibility factors might have a much higher risk of death such as the presence of chronic disease, heavy drinking habits, diabetes, malignancy, renal disease, delay of treatment, and health-compromising factors [5,9].

In the current study, we retrospectively analyzed the clinical data of patients with laboratory-confirmed VVI in our hospital and we tried to identify the independent predictors of mortality with the over-arching objective of improving the survival of these patients.

## Materials and Methods

### Patients

From January 2000 to October 2014, patients that presented with laboratory-confirmed VVI were enrolled in our hospital. The medical records were reviewed, and included analysis of the following: demographics, clinical characteristics, laboratory results, treatment history, the time between the onset of the condition, and arrival at our hospital, and hospitalization. All of the patients with laboratory-confirmed infections were divided into a survival group and a non-survival or death group according to their clinical outcomes (Details were shown in S1 Table).

### Ethics Statement

All the patients signed written informed consent, and the Ethics Committee of our hospital, the Medical ethics committee of the First Affiliated Hospital, College of Medicine, Zhejiang University, approved the study, which conformed to the ethical guidelines of the Helsinki Declaration.

### Diagnostic criteria

Mortality was defined as death during hospitalization or via telephone follow-up confirming death after being discharged from our hospital. Excessive drinking habits, was defined as more than one alcoholic beverage per day for women, and more than two alcoholic beverages per day for men[14]. Septic shock was diagnosed according to the guidelines that were established in 2001[15]. Phlegmon was defined as inflammation of the connective tissue, especially the subcutaneous connective tissue, which was usually suppurative.

### Statistical Analysis

Statistical Package for the Social Sciences version 16.0 for Windows (SPSS Inc., Chicago, IL, USA) was used for data analyses. Descriptive statistics are shown as mean ± standard deviation for continuous data and percentages for categorical data [9]. Between-group differences in continuous data were tested using the Mann–Whitney U test. Categorical variables were analyzed using either the Pearson χ^2^ test or Fisher’s exact test [9]. All tests for significance were two-sided, and *p* values < 0.05 were considered statistically significant.

## Results

### Epidemiology of VVIs

**Fig 1:**
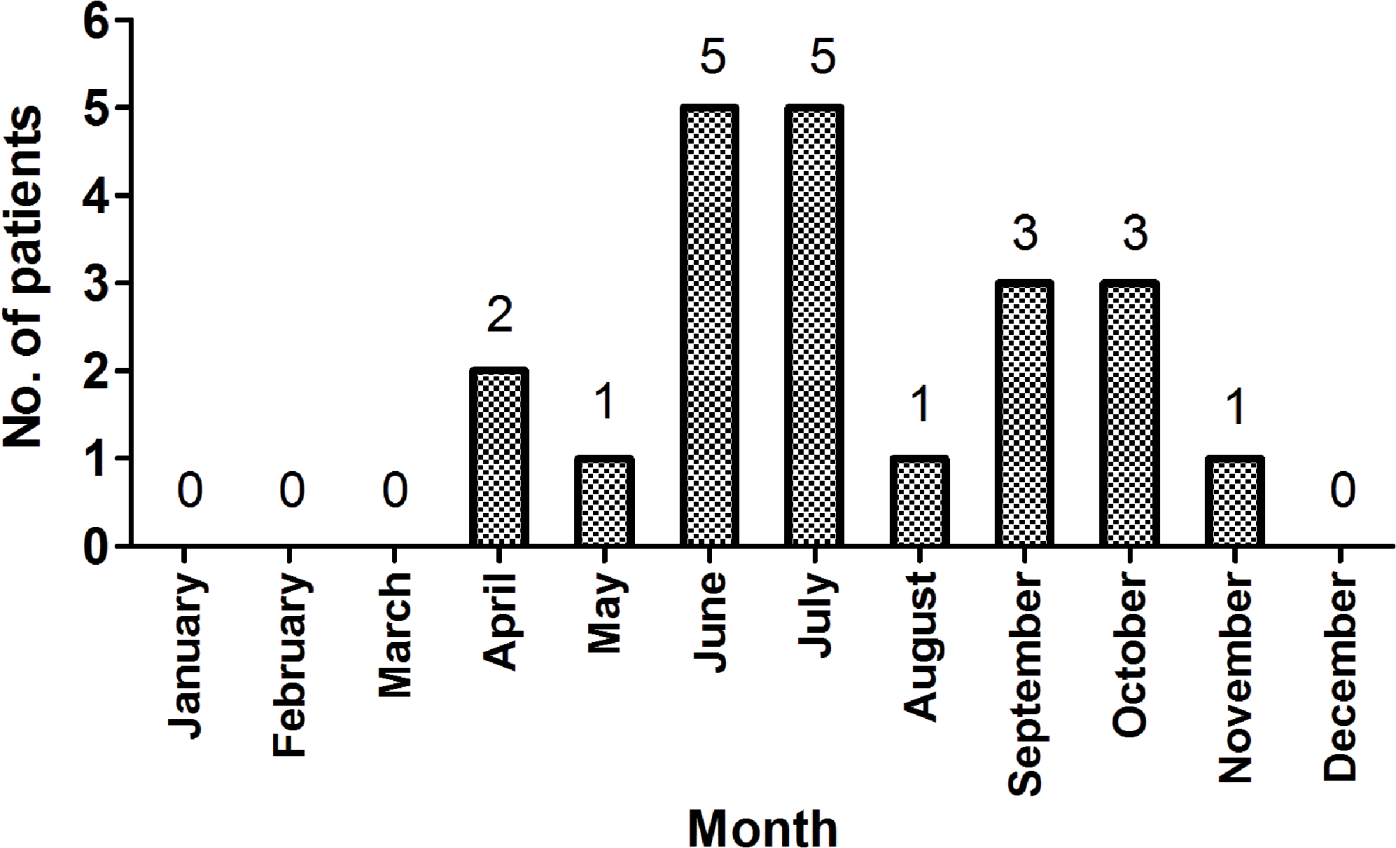
Seasonal distribution of *V*. *vulnificus* infections at the First Affiliated Hospital of Zhejiang University, School of Medicine, Hangzhou, China.

Twenty one patients were diagnosed with laboratory-confirmed VVIs at our hospital during the past 14 years. The majority of these patients (20/21, 95.2%) were infected between May and October (Fig 1), and all patients came from Southeast China.

### Demographics and clinical manifestations caused by *V*. *vulnificus*

**Fig 2:**
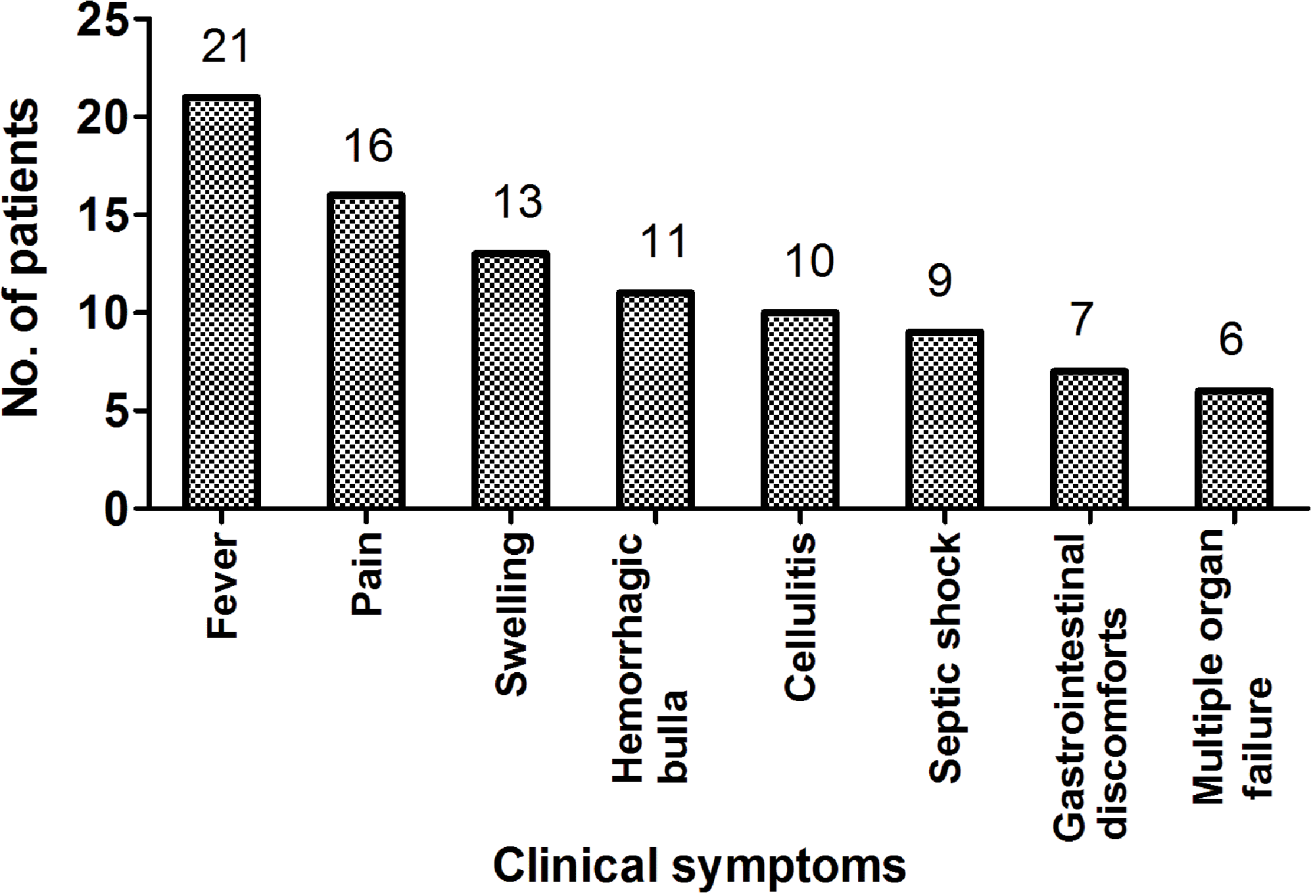
Clinical syndromes of *V*. *vulnificus* infections in the First Affiliated Hospital of Zhejiang University, School of Medicine, Hangzhou, China.

**Fig 3:**
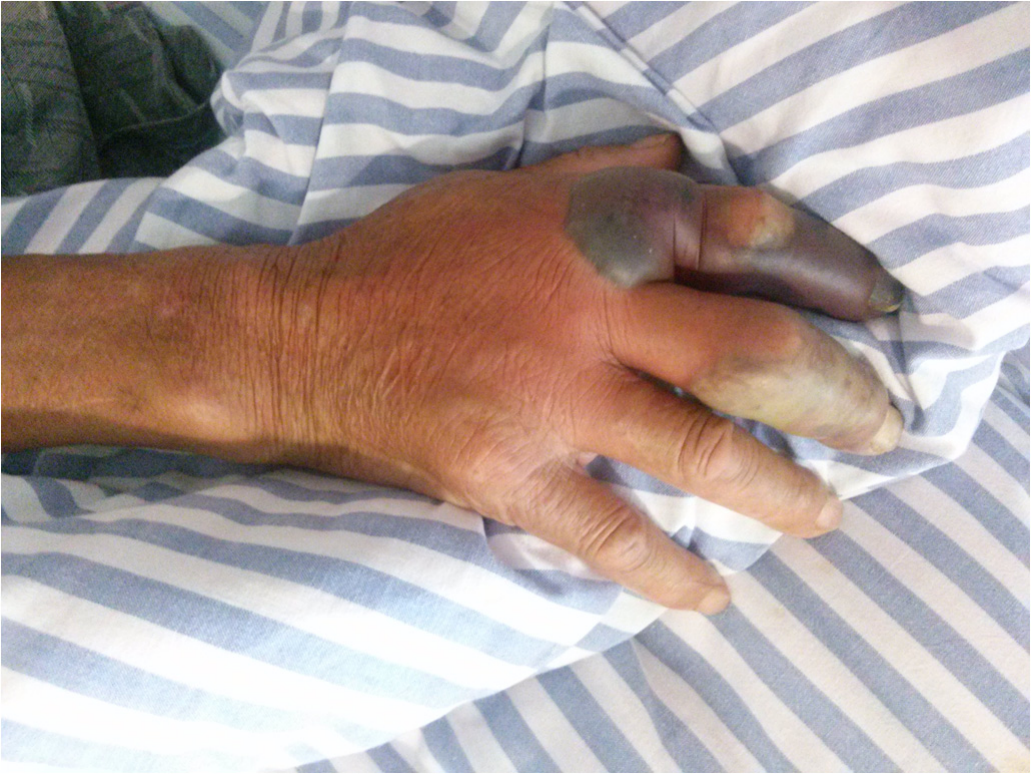
*V*. *vulnificus* infection that presented as swelling, Hemorrhagic bulla and phlegmon are observed on the right hand.

Sixteen of the 21 patients presented with evidence of soft tissue infections, of which eight were diagnosed with sepsis, and three had abdomen infections. Meanwhile, eight patients were confirmed by blister fluid culture, six patients were confirmed by blood culture, and five patients were confirmed by bile fluid culture. Fourteen of the patients were males and seven were women among the 21 recruited patients. The mean age was 54.57 ± 8.58 years (ranging from 39 to 68 years). Four patients died, and the mortality rate was 19.05%. Marine-related events were found in 16 patients (16/21, 76.2%) including 13 patients that were injured while handling marine animals or exposed to seawater, and three patients who had consumed seafood. Five patients had an unknown history of events. Also, two patients that did not present with any clinical manifestations that were caused by VVI were positive for *V*. *vulnificus* by bile fluid culture following cholecystectomy due to gallbladder stones. The most common clinical symptoms were fever (100%, 21/21) and pain (76.2%, 16/21). Hemorrhagic bullas were seen in 52.4% (11/21) and swellings were present in 61.9% (13/21) of patients. Higher mortality rates were seen in patients with cellulitis (47.6%, 10/21, p = 0.035, OR = 1.7, 95% CI 1.0–2.8), septic shock (42.9%, 9/21, p = 0.021, OR = 1.8, 95% CI 1.0–3.2) and multiple organ failure (28.6%, 6/21, p = 0.003, OR = 3.0, 95% CI 1.0–9.3) (Table 1, Fig 2, Fig 3).

**Table 1:** Demographic and clinical presentations.

Characteristic	Survivors (n = 17)	Deaths (n = 4)	OR(95%CI)	p-value
Age (years)				
Mean ± SD	54.82 ± 8.74	53.50 ± 9.00		0.789
Range	39–68	42–64		
Gender				
Male/female	10/7	4/0	0.7(0.5–1.0)	0.255
Blood type				
A/B/O/AB	4/4/6/3	2/2/0/0		0.091
Comorbidities				
None	4	1	0.9(0.1–11.5)	1.000
Heavy drinking	2	3	22.5(1.5–335.3)	0.028
Liver disease^&^	9	1	0.3(0.0–3.5)	0.586
Clinical symptoms				
Fever	17	4		
Pain	12	4	1.3(1.0–1.8)	0.532
Swelling	9	4	1.4(1.0–2.1)	0.131
Hemorrhagic bulla	9	2	0.9(0.1–7.9)	1.000
Cellulitis	6	4	1.7(1.0–2.8)	0.035
Gastrointestinal discomforts*	7	0	0.7(0.5–1.0)	0.255
Septic shock	5	4	1.8(1.0–3.2)	0.021
Multiple organ failure	2	4	3.0 (1.0–9.3)	0.003
Pathogens cultured site				
Bile/not bile culture	5/12	3/1	1.3(1.0–5.8)	0.532

Note

^&^: Chronic liver diseases include hepatitis B, and C, alcoholic hepatitis, liver cirrhosis, liver transplantation, and liver hepatic carcinoma.

*: Gastrointestinal discomfort including abdominal pain, vomiting, and diarrhea.

### Laboratory results of VVI

The laboratory results measured at the time the patients presented to our hospital (Table 2) and showed that hemoglobin levels < 9.0g/L (p = 0.012, OR = 12.6, 95%CI 11.8–92.7), platelets < 2.0×10^9^/L (p = 0.012, OR = 12.6, 95%CI 11.8–92.7), PTA < 20% (p = 0.003, OR = 18.0, 95%CI 2.7–121.0), and decreased serum creatinine (p = 0.028, OR = 9.6, 95%CI 1.3–73.0) and increased urea nitrogen (p = 0.028, OR = 9.6, 95%CI 1.3–73.0) all predicted a significantly higher mortality.

**Table 2:** Laboratory results measured between survivors group and deaths group.

Laboratory results	Survivors(n = 17)	Deaths (n = 4)	p-value
WBC (mean ± SD, ×10^9^/L)	6.87 ± 4.59	11.53 ± 9.65	0.158
> 10×10^9^/L	4	1	1.000
< 4×10^9^/L	5	1	1.000
Hemoglobin (mean ± SD, g/L)	111.94 ± 16.57	87.00 ± 8.98	0.010
< 90 g/L	1	3	0.012
Platelets	85.24 ± 61.65	8.75 ± 1.50	0.000
< 2.0×10^9^/L	1	3	0.012
PT (mean ± SD, s)	17.38 ± 4.85	42.30 ± 5.31	0.004
PTA<20%	0	3	0.003
Total bilirubin (mean ± SD, mmol/L)	95.58 ± 84.51	198.67 ± 169.67	0.165
>increased / normal	6/11	2/2	0.618
ALT (mean ± SD, U/L)	90.00 ± 94.35	169 ± 175.81	0.313
>2 times ULN/normal	6/11	1/3	1.000
AST (mean ± SD, U/L)	125.30±91.06	814.00 ± 1045.36	0.372
>2 times ULN/ normal	6/11	3/1	0.272
Serum creatinine (mean ± SD, μmol/L)	83.30 ± 50.95	375.00 ± 257.46	0.187
< normal / normal	2/15	3/1	0.028
Urea nitrogen (mean ± SD, mmol/L)	7.044 ± 4.07	34.96 ± 16.97	0.182
≥normal / normal	2/15	3/1	0.028

Note: ULN, the upper limit of normal. At our hospital, the normal range for ALT was 5–40 U/L, the normal range for AST was 5–37 U/L, the normal range for serum creatinine was 40–108 μmol/L, and the normal range for urea nitrogen was 1.7–8.3 mmol/L.

These clinical isolates were tested for susceptibility to meropenem or imipenem, piperacillin, piperacillin/tazobactam, cefepime, cefoperazone/sulbactam, cefotaxime, ceftazidime, ciprofloxacin, cotrimoxazole, levofloxacin. One bacterial isolate was resistant to imipenem, piperacillin/tazobactam, cefoperazone/sulbactam, cefotaxime, ceftazidime, ciprofloxacin, cotrimoxazole, and levofloxacin. However, it was sensitive to tigecycline.

### Treatment strategies and clinical outcomes in patients with *V*. *vulnificus*

The time of treatment, hospitalization, antibiotics strategies, the course of treatment, and mortality seen between the survival group and the non-survival group are summarized in Table 3.

**Table 3:** Treatment strategies and clinical outcomes.

Treatment strategies	Survivors(n = 17)	Deaths (n = 4)	p-value
Antibiotic treatments			0.272
Only on antibiotic	6	3	
Piperacillin-tazobactam	2	0	
Cefoperazone-sulbactam	1	2	
Imipenem-cilastatin	2	0	
Meropenem	0	1	
Quinolone	1	0	
More than one antibiotic	7	1	
Surgical interventions			
Yes/No	5/12	0/4	0.532
The time between the injuries or the onset of symptoms to hospital (mean ± SD, day)	2.29±2.34	2.25±0.96	0.971
<one />one	10/7	3/1	1.000
Range	1–10	1–3	
Hospitalization (mean ± SD, day)	39.71±32.49	15.00±8.72	0.155
Range	7–109	2–20	

The mean time between injuries or the onset of symptoms to hospitalization in the survival group was 2.29 ± 2.34 days (ranging from 1 to 3 days) and 2.25 ± 0.96 days (ranging from 1 to 10 days) in the non-survival group (t = 0.036, p = 0.971). The mean time of hospitalization was 39.71 ± 32.49 days (ranging from 7 to 109 days) in the survival group and 15.00 ± 8.72 days (ranging from 2 to 20 days) in the non-survival group (t = 1.481, p = 0.155).

All patients used antibiotics after admission, and six patients used only one antibiotic and 11 patients used more than one antibiotic in the survival group as compared with the non-survival group where there were three patients that used only one antibiotic, and one patient used more than one antibiotic (p = 1.000). There was no significant difference seen in survival outcome between patients that underwent surgery and those patients that did not undergo surgery (p = 0.532).

## Discussion

*V*. *vulnificus* is found worldwide in warm coastal waters. Infections with *V*. *vulnificus* are commonly fatal, and the speed and accuracy of diagnosis and treatment is directly linked to mortality [5]. At our hospital, the 19.05% mortality was less than most that were previously reported, and the decreased mortality can be explained due to early diagnosis and appropriate treatment [12,15]. First, all patients came from the Southeast of China and most of these patients (20/21, 95.2%) were infected during the periods of May to October. Meanwhile, 76.2% (16/21) of the patients presented with evidence of soft tissue infections and 76.2% (16/21) of these patients had a positive history of marine-related events. All of these clinical findings allowed us to make an early diagnosis. Second, all of our patients were treated with antibiotics at our hospital. Most patients chose third-generation cephalosporin or the penicillin compound preparation, carbapenemin. In addition, some of the patients chose one of those antibiotics plus tetracycline, and those antibiotics had proven effective against VVIs [5].

One of the more interesting outcomes from our study is that in our tests, there were two patients who did not have any clinical manifestation caused by VVI, and yet these patients were bile fluid culture positive for *V*. *vulnificus* after cholecystectomy, due in part to gallbladder stones. We may safely conclude that *V*. *vulnificus* may actually become an invisible infection, and the pathogens could lurk undetected *in vivo* (e.g., in the gallbladder as we had demonstrated). It is possible for human subjects to be infected when the body's immunological resistance is dampened or transiently immuno-compromised [5].

Some high-risk patients may be infected following consumption or from handling contaminated seafood or exposing open wounds to saltwater, such as chronic liver disease, immunodeficiency, end-stage renal disease, alcoholism, and diabetes mellitus, among others [5,9,10,13,16]. In our present study, those who are also suffering from chronic liver disease and diabetes mellitus did not affect the patients’ survival rate, while habitual heavy drinking affected the overall survival rate of patients with VVI.

Some studies have identified clinical indicators for the early diagnosis and surgical treatment of VVIs. Hong *et al*. found the time from first presentation of symptoms to hospital admission and limb lesions that involved the trunk might affect the prognosis of the patient [10]. Lee *et al*. found that the number of operations will affect survival [9]. In our series, however, we found that the time between injuries or the first presentation of symptoms when appearing at the hospital, and the appearance of hemorrhagic bulla, did not affect the prognosis of the patient.

We believe that the number of patients who died or had undergone surgical operations was less than others had reported. Additionally, most patients were treated with antibiotics before arriving at our hospital, and the real time from appearance of symptoms to hospital admission might be less than our statistical time. Similar to other previous studies, the time of hospitalization between the survival and non-survival groups was very similar [9,10]. From our limited set of data, we might infer that the time of hospitalization for the non-survival group was less than the survival group, but this conclusion has to be clearly demonstrated in a larger number of patients.

Tsai *et al*. statistically analyzed patients that presented with Vibrio necrotizing fasciitis and revealed that a systolic blood pressure of 90 mmHg, low platelet counts, and a combination of hepatic dysfunction were associated with a higher mortality rate [16]. In addition, Chou *et al*. studied 119 adult patients that were hospitalized for VVIs, and their statistical analysis revealed that a high degree of organ dysfunction or a high infection score (or both) were associated with mortality [11].

In our study, we found in some patients that cellulitis, septic shock and multiple organ failure will all affect mortality (p = 0.035, OR = 1.7, 95%CI 1.0–2.8; p = 0.021, OR = 1.8, 95%CI 1.0–3.2; and p = 0.003, OR = 3.0, 95%CI 1.0–9.3 respectively). Meanwhile, adverse laboratory results also affected mortality in some patients and these included hemoglobin < 9.0g/L (p = 0.012, OR = 12.6, 95%CI 11.8–92.7), platelets < 2.0×10^9^/L (p = 0.012, OR = 12.6, 95%CI 11.8–92.7), PTA < 20% (p = 0.003, OR = 18.0, 95%CI 2.7–121.0), decreased serum creatinine (p = 0.028, OR = 9.6, 95%CI 1.3–73.0) and a urea nitrogen that is increased (p = 0.028, OR = 9.6, 95%CI 1.3–73.0). Cellulitis affects survival, as has been previously reported by Hong *et al*. [10]. In addition, in laboratory tests, PTA < 20% represented serious hepatic dysfunction. Moreover, under conditions of decreased serum creatinine and increased urea nitrogen, these might be associated with renal dysfunction. All of these laboratory findings revealed multiple organ dysfunction. Meanwhile, low platelet counts (< 2.0×10^9^/L), and decreased hemoglobin (< 9.0 g/L) were consistent with previously reported other studies [11].

In conclusion, some patients with risk factors consistent with susceptibility to VVI, and who have acute sepsis, fever, cellulitis, and skin hemorrhagic bullae, should be highly suspected of showing VVI, especially in the summer or autumn seasons. Patients may be at a higher risk of mortality under situations where they have a history of habitual heavy alcoholic drink consumption, accompanied with cellulitis, shock, multiple organ failure, and laboratory examinations that are complicated by decreased platelets, hemoglobin and significantly prolonged PT. To prevent VVI, we have to enhance health awareness, and individuals have to avoid consumption of raw seafood, be aware of the risks of damaged skin or open wounds, or mucosal injury, avoid contact with or submersion in sea water. Early diagnosis and selection of appropriate antibiotics are critical factors in attempts aimed at saving the patient's life.

## Supporting Information

S1 Table Descriptive data of subjects in the study. (XLS)
